# Comparison of Single- vs. Dual-Lead CIEDs Regarding Perioperative Complications-Analysis of the Quality Assurance Data of the State of North Rhine-Westphalia, Germany

**DOI:** 10.3390/jcm14093203

**Published:** 2025-05-06

**Authors:** Marvin Scholten, Sotirios Nedios, Zana Karosiene, Fuad Hasan, Bernd Lemke, Heinz Jürgen Adomeit, Stephanie Knippschild, Markus Zarse, Harilaos Bogossian

**Affiliations:** 1 Department of Anaesthesiology, Intensive Care Medicine and Emergency Medicine, Evangelisches Klinikum Niederrhein, 47169 Duisburg, Germany; 2 Department of Cardiology, University Witten/Herdecke, 58455 Witten, Germany; 3 Department of Cardiology, Herzzentrum Leipzig-University Leipzig, 04289 Leipzig, Germany; 4 Department of Cardiology, Electrophysiology, and Angiology, Klinikum Luedenscheid, 58515 Luedenscheid, Germanybernd.lemke@klinikum-luedenscheid.de (B.L.); 5 Medical Quality Management, Head of the Quality Management Office QS-NRW, Regional Representation Westphalia-Lippe, 48147 Münster, Germany; 6 Faculty of Health, Institute for Medical Biometry and Epidemiology, Witten/Herdecke University, 58455 Witten, Germany; 7 Department of Cardiology and Rhythmology, Evangelisches Krankenhaus Hagen-Haspe, 58135 Hagen, Germany

**Keywords:** pacemaker implantation, ICD implantation, perioperative complications, independent risk factors, single vs. dual lead devices, mortality, big data analyzes

## Abstract

**Background/Objective:** Previous studies have indicated a higher incidence of complications associated with dual-lead devices compared to single-lead devices. This retrospective study aimed to investigate the effect of lead count and other factors on peri/postoperative complications for new pacemaker (PM) or implantable cardioverter-defibrillator (ICD) implantations in a representative German cohort. **Methods:** In accordance with quality assurance (QS) requirements, all hospitals in Germany collect patient-specific data on cardiac device implantation. We utilized the QS database from North Rhine-Westphalia to analyze data from 169,547 patients who underwent PM (*n* = 137,208) or ICD (*n* = 32,339) implantation between 2010 and 2014. The primary endpoint was the incidence of perioperative complications in the PM and ICD groups, analyzed separately for single- and dual-lead systems. Regression analysis was performed to identify specific risk factors and the effect of lead number on complications. **Results:** In the PM group, patients with dual-lead devices experienced significantly more complications than those with single-lead devices (3.27% vs. 2.85%, *p* < 0.001), largely driven by lead dislocations. In the ICD group, no significant difference in complication rates was observed between single-lead and dual-lead devices (1.39% vs. 1.46%, *p* = 0.635). The risk of complications was over twice as high for PM implantation compared to ICD implantation (3.17% vs. 1.42%). Patients with intrahospital complications faced a significantly higher risk of mortality than those without complications (PM: 4.5% vs. 1.6%; ICD: 3.9% vs. 0.5%). Independent risk factors for complications and mortality were identified in both groups, with a notable increase in the risk of intrahospital mortality associated with complications. **Conclusions:** Complications of dual-lead PM implantations are higher than single-lead procedures, mostly due to lead dislocations. Such a difference, though, was not observed in ICD implantations. Our findings may help optimize procedural strategies to improve patient safety, especially in PM implantations.

## 1. Introduction

Pacemaker (PM) and implantable cardioverter-defibrillator (ICD) implantations are generally safe procedures with rare complications; however, the mortality risk of such complications may be underestimated.

Germany is a country with a high volume of PM/ICD implantations that are all subject to a quality assurance (QS) registry. All hospitals in Germany collect patient-specific data on cardiac device implantation. This paucity of data can reach up to 93,806 implantations per year (2020) that are associated with the respective codes (PM: 72,489; ICD: 21,317) and the obligatory registration of peri/postoperative complications in order to achieve quality assurance (QS) standards [1,2]. 

The number of leads (dual vs. single) has been previously recognized as a strong risk factor for complications, but this has never been investigated separately for PMs and ICDs. Thus, this study aimed to examine a large cohort from a German federal state for the effect of lead count or other factors on complications of PM and ICD implantations.

## 2. Materials and Methods

**Figure 1:**
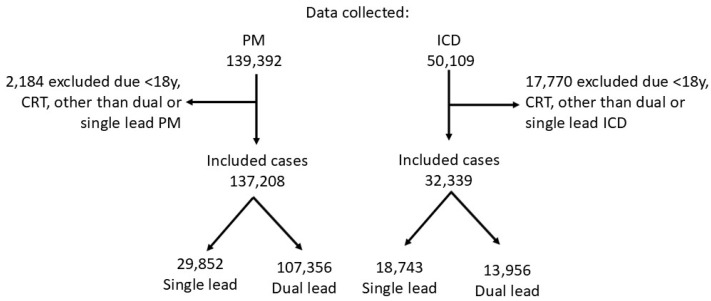
Study protocol.

In Germany, all hospitals are legally required (SGB V) to collect patient-specific data for quality assurance (QS) related to cardiac device implantation. The QS database of North Rhine-Westphalia (NRW) recorded all patients who underwent new pacemaker (PM) or implantable cardioverter-defibrillator (ICD) implantations between 2010 and 2014 (*n* = 189,501). Data for PM and ICD procedures were stored separately in two databases. The chosen time period (2010–2014) was determined based on data availability, as no other data were accessible for analysis at the time due to regulatory restrictions. After excluding patients under 18 and those with device types other than dual- and single-lead, 169,547 patients remained for analysis. During the study period, 137,208 dual- or single-lead PMs and 32,339 dual- or single-lead ICDs were implanted in NRW and included in this retrospective case-control study (Figure 1). 

PM and ICD data were analyzed separately due to their differing indications. The primary endpoint was the occurrence of perioperative intrahospital complications, defined as pneumothorax, hemothorax, asystole, ventricular fibrillation (VF), cardiopulmonary resuscitation (CPR), pericardial effusion or tamponade, lead dislocation or dysfunction, hematoma, wound infection, or other.

To address the analysis of the PM and ICD populations and the issue of multiple testing within the same study, we conducted the Bonferroni correction (α = 5/2% = 2.5%) for the central research question. The adjusted *p*-value is *p* = 0.025. The data analysis was conducted retrospectively using IBM® SPSS® Statistics Version 27 (Version 27.0.0.0, 64-bit; SPSS Inc., Chicago, IL, USA) for Windows and Microsoft® Excel® for Microsoft 365 MSO (32-bit). Primary statistics were performed using Fisher’s test and the Chi^2^-test. Variables that showed a significant difference between groups in these tests or had a previously established relevant influence were included in an univariable logistic regression model. Subsequently, a multivariable logistic regression model was applied to identify independent risk factors for perioperative intrahospital complications for the PM and ICD subgroups. The results of the multivariable analysis were adjusted in PM-Group for gender, age, LVEF, PM system (lead count), ASA classification, indication for implantation, atrial rhythm, and AV and ventricular conduction. For the ICD Group, the results of the multivariable analysis were adjusted for gender, age, LVEF, ICD system (lead count), ASA classification, indication for implantation, atrial rhythm, and AV and ventricular conduction. The identified groups are described with their Odds Ratio (OR), 95% Confidence Intervals (CIs), and separate *p*-values.

## 3. Results

### 3.1. Study Population

During the study period, a total of 189,501 patients underwent a new cardiac implantable electronic device (CIED) implantation procedure. Patients under the age of 18 and those with device types other than single- or dual-lead pacemakers (PM) or implantable cardioverter-defibrillators (ICD) were excluded (*n* = 19,954). The final study population comprised 137,208 PM patients and 32,339 ICD patients.

### 3.2. Patient and Procedural Characteristics

In the PM group, most patients underwent dual-lead PM implantation (*n* = 107,356; 78.2%). The median age at implantation was 78 years (interquartile range [IQR]: 72–84), with a slight predominance of men (51.9%). Among single-lead PM recipients, VVI-PMs were the most common (*n* = 29,029), whereas AAI (*n* = 197) and VDD (*n* = 626) devices were less frequently used. Over 90% of PM recipients had mild to severe systemic disease (ASA II–III), as defined by the American Society of Anesthesiologists physical status classification, and most showed a normal left ventricular ejection fraction (LVEF > 50%). The most common clinical symptom leading to PM implantation was presyncope and dizziness (46%), followed by recurrent syncope (27.9%) and single syncope (12.3%). The leading indication for PM implantation was sick sinus syndrome or bradycardia-tachycardia syndrome (40.8%), followed by third-degree atrioventricular block (26.0%) and bradycardic atrial fibrillation (16.7%). No differentiation was made regarding the indication for implantation between single- and dual-lead PM (Supplementary Table S1).

In the ICD group, single-lead ICDs were more frequently implanted (*n* = 18,743; 58%). The median age was 69 years (IQR 59–75), with men comprising the majority of recipients (78.2%). VVI-ICDs were the most common device type among single-lead ICD patients (*n* = 18,743), while VDD-ICDs were less common (*n* = 399). Most ICD patients had severe systemic disease (ASA III) and had an LVEF under 35%, with the majority presenting with heart failure symptoms NYHA Class II or III. Among ICD recipients, 65% received the device for primary prevention and 34.2% for secondary prevention. In the latter group, the index clinical events included ventricular fibrillation (14.6%), sustained ventricular tachycardia (11.8%), and non-sustained ventricular tachycardia (6.5%). The most frequent presenting clinical events in these patients were cardiac arrest with resuscitation (16.2%) and syncope (8.7%). No differentiation was made regarding the indication for implantation between single- and dual-lead ICD (Supplementary Table S2).

### 3.3. Complications

In the PM group, patients with an implanted dual-lead device had significantly more complications (3.27% vs. 2.85%, *p*_/2_ < 0.001) than single-lead devices (Table 1). In the ICD group, there were no significant differences in the occurrence of complications (1.39% vs. 1.46%, *p*_/2_ = 0.635) between single-lead and dual-lead ICDs (Table 2). Therefore, the complication risk of PM implantation was more than twice as high as that of an ICD (3.17% vs. 1.42%).

Lead dislocations were the most common complication in both groups (Table 3 and Table 4). Dual-lead PMs experienced more lead dislocations than single-lead PMs, primarily due to a higher incidence of atrial lead dislocations. In contrast, single-lead PM (AAI & VVI) had a higher rate of ventricular lead dislocations and no atrial lead dislocations. No significant difference in lead dislocations was observed within the ICD group. Single-lead PMs showed a significantly higher incidence of pocket hematomas, and single-lead ICDs had more pocket hematomas as well as a higher rate of hemothorax. Intrahospital perioperative infections for both PMs and ICDs were rare, with no significant difference between single- and dual-lead devices.

### 3.4. Complication Risk

In the PM group, women had a relatively high incidence of perioperative complications (3.6%). The highest complication rate was observed in patients aged 20–29 years (11.1%), with elevated rates also seen in those aged 50–89 years. Among patients with systemic disease, those with mild conditions had an incidence of 3.5%, severe cases had 2.8%, and severe, life-threatening conditions showed a rate of 4.6%. High complication rates were also noted in patients with complete left bundle branch block (LBBB), congenital heart disease, neuromuscular disease, and carotid sinus syndrome. Conversely, lower-than-average complication rates were observed in those with second-degree atrioventricular block (AVB II; Mobitz I), incomplete LBBB, alternating bundle branch block, and bradycardic atrial fibrillation.

In the ICD group, women, older patients, and those with a low LVEF exhibited a higher incidence of perioperative complications. The highest complication rate was observed in patients receiving a VDD-ICD (7.6%). Patients without diabetes mellitus had a slightly higher incidence of perioperative complications, as did those with renal insufficiency who were not undergoing renal replacement therapy. Additionally, patients with left or right bundle branch block had a higher complication rate, whereas those with first-degree atrioventricular block (AVB I) did not.

### 3.5. Complication Predictors

**Figure 2:**
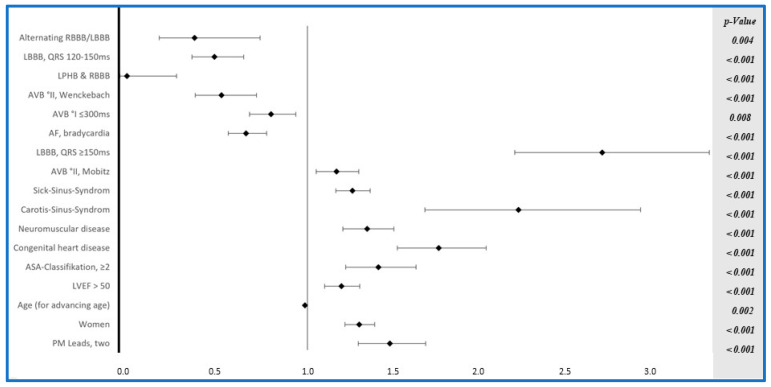
Forrest Plot of multivariable logistic regression for PM.

In both the PM and ICD groups, independent risk factors were identified as female sex and the presence of LBBB. Additionally, in the PM group, further independent risk factors for intrahospital complications included young age, congenital heart disease, carotid sinus syndrome, sick sinus syndrome, ASA classification ≥ 2, and the use of dual-lead PMs. On the other hand, alternating RBBB/LBBB, LBBB (120–150 ms), AVB°I and AVB°II (Wenckebach), and bradycardia in AF decrease the risk of a complication (Figure 2).

**Figure 3:**
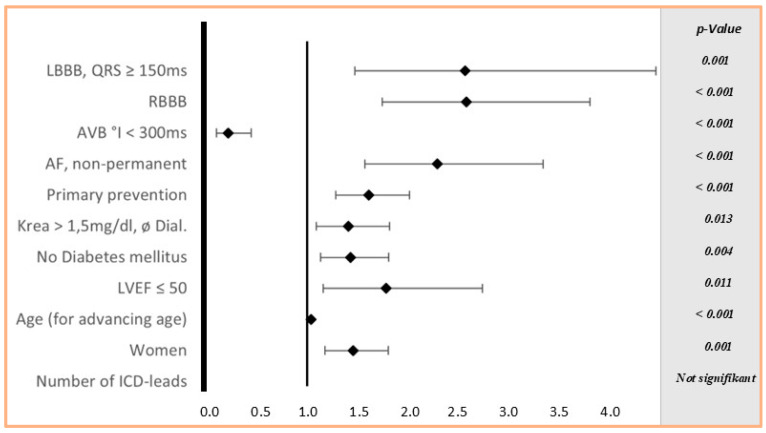
Forrest Plot of multivariable logistic regression for ICD.

In the ICD group, independent risk factors for complications included advanced age, left ventricular ejection fraction (LVEF) ≤ 50%, absence of diabetes, renal insufficiency without renal replacement therapy, primary prevention indication, and non-permanent atrial fibrillation (Figure 3).

### 3.6. Mortality

Patients who experienced an intrahospital complication also exhibited a heightened risk of intrahospital mortality in both groups (PM: 4.5% vs. 1.6%; ICD: 3.9% vs. 0.5%). Mortality among PM patients who experienced a complication was three times higher compared to those without complications (Table 5), whereas in the ICD group, mortality was eight times higher among patients with complications compared to those without (Table 6).

## 4. Discussion

### 4.1. Complication Risk

The present study investigated complication rates for pacemaker (PM) and implantable cardioverter-defibrillator (ICD) implantation. Our study found a complication rate of 4.4% for PM and only 1.4% for ICD. Dual-lead PMs were associated with significantly more complications than single-lead PMs (3.27% vs. 2.85%, *p*_/2_ < 0.001), while no significant difference was observed between ICD types (1.39% vs. 1.46%, *p*_/2_ = 0.635). This is comparatively lower than previously published data, where complication rates for PM implantation range from 4% to 12.4% [3,4,5,6,7,8,9,10,11,12], with one study reporting as low as 2.5% [13]. For single-lead PM, the incidence of complications ranges from 2.5% to 7.7%, while for dual-lead PM it ranges from 4.4% to 10.4% [3,4,9,14,15,16,17]. The ICD complication rates vary from 1% to 19% [18,19,20,21,22,23,24,25,26,27,28,29,30,31,32,33,34,35,36,37], with single-lead ICDs having an incidence from 1.1% to 8.8% and dual-lead ICDs ranging from 1.6% to 16% [15,21,38,39,40,41,42,43,44,45]. This discrepancy between the data from our study and other literature may be attributed to the inclusive nature of the quality assurance registry and the relatively short follow-up period, which was limited to the index hospitalization. Interestingly, in our study, dual-lead PM was associated with significantly more complications than single-lead PM, while this was not observed in the ICD group. This discrepancy could be explained by the different indications for lead selection, as the number of leads required is often dependent on the underlying disease or rhythm [46]. While anti-bradycardic stimulation may occasionally be necessary in patients with ICD, PM indications, particularly in cases of AV block, typically require systems with two leads [47,48]. In our study, 78.2% of PM were implanted with DDD-PM, whereas only 42.6% of ICD were implanted as DDD-ICD. We hypothesize that the number of implanted leads could be a contributing factor to the increased risk of complications in dual-lead devices. The distribution between single-lead and dual-lead ICDs must be taken into account and could be a potential reason why no difference is observed between single- and dual-lead ICD systems. Similarly, Chauhan et al. (*n* = 2019) reported a higher complication rate at 6 weeks after a dual-lead than after a single-lead PM (8.7% vs. 2.9%, *p* < 0.05) from 1983 to 1992. This was attributed to higher rates of infections and lead dislocations in the dual-lead group (5.2% vs. 1.0%). The incidence of atrial or ventricular lead dislocation was 3.8% and 1.4%, respectively. Since single-lead implants were more common than dual-lead PM, operator experience (3.2 DDD-PM per year/surgeon) may have contributed to the findings [3]. Eberhardt et al. also demonstrated the impact of experience, reporting a significantly higher complication rate in patients implanted by surgeons with low to moderate experience, while no such increase was observed with highly experienced surgeons [15]. Tobin et al. also showed an inverse correlation between complication rate and number of implantations per year. The incidence of lead dislocations decreased with increasing number of procedures but not with surgeon experience [49]. Thus, the need for an experienced operator in dual-chamber PM should not be underestimated. 

We also found a statistically significant difference in the occurrence of pocket hematomas between single-lead and dual-lead PM, as well as ICD implantations. Single-lead devices are often indicated for patients with atrial fibrillation who should receive therapeutic anticoagulation and therefore have an increased risk of bleeding [49,50,51]. Dual therapy with acetylsalicylic acid and thienopyridines (ticlopidine and clopidogrel) following coronary stent placement, as well as limited operator experience, were also identified as independent predictors of hematoma formation [51]. The training of operators typically begins with the implantation of simpler devices like single-lead PM [49,50]. Pakarinen et al. found in their study that pocket hematomas were more common among trainees (5.0%) compared to fully trained cardiologists (1.8%, *p* = 0.037) [50].

Additionally, other risk factors for complications, such as age and gender, were identified in our study. Patients aged 20–29 had the highest incidence of perioperative complications (11.1%, *n* = 34), with this subgroup commonly presenting with congenital heart disease, which is consistent with findings in the literature (10.6%) [12]. Women also have a higher incidence of complications, which has been reported in previous studies [52,53,54], independent of age or PM type [52]. Anatomical differences such as smaller vessel diameter, smaller thoracic cavity, thinner right ventricular wall, and smaller diameter of the coronary sinus have been cited as causes for higher rates of thoracic and cardiac complications in women [55]. Furthermore, women have fewer complications in hospitals with high implantation volumes [56].

For ICDs, only two studies found no difference in complication incidence between single- and dual-lead ICDs [38,45], while several other studies reported significant differences [21,31,41,42,44,57,58]. The two studies that showed no difference between single- and dual-lead complication rates were only small and methodologically weak. Dewland et al. reported more complications in dual- than in single-chamber ICDs (3.17% vs. 2.11%), but also included complications such as myocardial infarction, stroke, or drug side effects [41].

The highest incidence of intrahospital complication was observed in patients receiving a VDD-ICD (7.8%). This contrasts with other studies where the incidence was lower than in DDD-ICD but higher than in VVI-ICD (6.3% vs. 3.2% vs. 2.6%) [15]. In the past, it was assumed that VVI and VDD systems had a similar learning curve and were suitable for surgeons with low experience seeking the advantages of a dual-lead device for AV synchrony [15]. Despite challenges in the modern pacing era, VDD systems may offer advantages such as shorter implantation times and lower complication rates and remain a viable option for selected patients [59].

Women also had a higher incidence of a complication than men [28,31,60,61,62], and it is assumed that similar reasons as with PM implantation contribute to this difference.

While our findings regarding the implantation of single- vs. dual-lead pacemakers and ICDs are important, it is clear that device programming may significantly impact right ventricular pacing and clinical outcomes such as heart failure and mortality [63]. However, due to the study design, programming was not considered, and the focus was limited to complications related to the implantation process.

### 4.2. Complication Predictors

We identified predictors of complications in both PM and ICD implantation, which may aid in device selection and the identification of high-risk patients. Our data show that in both the PM and ICD groups, female sex and the presence of LBBB were independent risk factors for in-hospital complications. In the PM group, additional predictors included younger age, congenital heart disease, carotid sinus syndrome, sick sinus syndrome, ASA classification ≥ II, and the use of dual-lead devices. Conversely, alternating RBBB/LBBB, LBBB with a QRS duration of 120–150 ms, AV block I/II (Wenckebach), and bradycardia in atrial fibrillation were associated with a lower risk. In the ICD group, risk factors included older age, LVEF ≤ 50%, absence of diabetes, renal insufficiency without dialysis, primary prevention, and non-permanent atrial fibrillation.

The FOLLOWPACE study identified the female gender as an independent risk factor, while men showed a risk reduction (HR 0.72; 95% KI: 0.53–0.97, *p* = 0.03) [10]. The registry data could also identify a dual-lead device as a risk factor for intrahospital complication, but not in the first two months. Additionally, our analyzed data showed risk reduction for older patients. Other possible factors are body weight (BMI), heart insufficiency, diabetes, anemia, liver disease, steroid medication, or a temporal pacemaker [10,11,64,65], but these data were not recorded in our data. Another significant factor could be the implantation technique itself. The implantation approach via cephalic vein cutdown (CVC) had significantly fewer perioperative complications than the subclavian puncture (SP) approach (2.49% vs. 3.64%, *p* = 0.0001, OR 1.47; 95% CI 1.38–1.57) [66].

The data about predictors of perioperative complications in ICD patients are limited. Haines et al. (2006–2008, *n* = 267,701) identified patients over 70 years old, previous heart valve surgery, chronic pulmonary disease, and heart insufficiency (NYHA III–IV) and also another reason for hospital admission [25]. Although age was not found to be a risk factor in other studies [31], this was the case in our study. Lee et al. also described non-ischemic cardiomyopathy and non-ischemic heart disease and anticoagulation therapy with ASS and Clopidogrel with a higher hazard ratio for complications. In this study also, patients with a dual-lead ICD had a higher risk (HR 1.82) and CRT (HR 2.17) in relation to a single-lead device [31]. Most of the complications were lead dislodgements. Cheng et al. identified in a multivariate analysis atrial fibrillation/flutter, NYHA IV, and CRT implantation as risk factors [39]. 

Risk reduction is achievable with a higher volume of ICD implantations, since increased experience correlates with lower mortality rates. Physicians in the upper quartile of procedure volume treated older and sicker heart failure patients and yet reported lower complication rates (2.9% vs. 4.6%) and mortality (0.36% vs. 0.72%) than those in the lower quartile [42]. Physician qualification (board certification/board eligibility in electrophysiology) is also important in reducing the risk of acute lead dislocations. Teaching/training hospitals were not found to be a risk factor (*p* = 0.64) for acute lead dislocation [39]. Kirkfeldt et al. also described procedure-related independent risk factors in CIED implantation for perioperative complications, such as centers with less than 750 implantations per year, low-volume operators (<50), and emergency or out-of-hours operations. Patient-related risk factors include female gender and underweight [53].

### 4.3. Mortality

PM implantations are generally considered safe procedures with low mortality rates. In our cohort, the overall mortality rate among PM patients was 1.7%; however, mortality among those who experienced a complication was three times higher compared to PM patients without complications (4.5% vs. 1.6%). Nowak et al. (Hessen, Germany, 2009, *n* = 5079) reported a mortality rate similar to our findings (1.5%). The complication rate among patients who died was also comparable (Hessen: 7.5% vs. NRW: 8.5%) [67]. A recent study from the United States (*n* = 242,980, 2016–2017) reported a lower mortality rate compared to our data (1.05% vs. 1.7%) [64]. It is important to note that mortality is not directly attributed to the procedure itself [67], but rather influenced by comorbidities [68]. 

In our cohort, the overall mortality rate among ICD patients was 0.5%. Notably, in patients who experienced a complication, mortality increased substantially to 3.9%, corresponding to an almost eight-fold higher rate compared to those without complications. In ICD patients, Bogossian et al. analyzed QS-NRW data (2010–2012, including CRT) and found mortality rates similar to our results among patients with and without complications (3.7% vs. 0.6%, *p* < 0.001). This study identified patients over 80 years old, those with heart failure (higher NYHA class), and those who received the ICD for secondary prophylaxis as at higher risk [69]. Conversely, Chen et al. observed a difference in mortality between single- and dual-lead ICD in non-randomized trials (RR 1.54; 95% CI, 1.29–1.84; *p* < 0.001), but not in randomized trials (RR, 1.04; 95% CI, 0.75–1.44; *p* = 0.81) [57].

While it is essential to consider both perioperative and long-term mortality, Imberti et al. a relatively small study (*n* = 838, PM *n* = 569, ICD/CRT *n* = 269) reported that 25.5% of patients with cardiac implantable electronic devices (CIEDs) died during a median follow-up of 3.5 years. Only age, a history of atrial fibrillation (AF), and end-stage chronic kidney disease (CKD) requiring dialysis were independently associated with all-cause mortality. No significant difference in mortality was observed between PM and ICD/CRT patients. This may be explained by the fact that ICD/CRT patients, although younger, have a significantly higher burden of comorbidities, whereas PM patients are considerably older [70]. These findings are consistent with the data from our study (see Supplementary Materials).

## 5. Study Limitations

One of the main strengths of this study is its presentation of “real-world data” from all hospitals in North Rhine-Westphalia (NRW) involved in new pacemaker (PM) and implantable cardioverter-defibrillator (ICD) implantations. The registry encompasses both high- and low-volume implantation centers and does not impose specific exclusion or inclusion criteria beyond those typically found in prospective studies. Each participating hospital followed standardized data submission protocols, contributing to the high quality of the data collected. This structured dialogue serves as a valuable quality tool, which may have financial implications for the hospitals involved.

However, like any retrospective registry study, our research has limitations. While these large data registries are part of an obligatory external quality assessment service, the quality of the submitted data relies heavily on the expertise, accuracy, and diligence of the submitters. Additionally, it is important to note that the reported complication rate may be underestimated, as only complications requiring intervention or therapy were required to be submitted, potentially omitting other relevant adverse events. The data used are from 2010–2014 and may not necessarily reflect recent advancements in lead implantation techniques, such as the increased use of conduction system pacing and the shift from dual-coil to single-coil ICDs. These technological innovations may potentially impact perioperative complication rates. However, a large portion of implantation practices remained unchanged. Nonetheless, these advancements should be considered in future studies to better align the findings with contemporary clinical practice. Another limitation of our study is the lack of data on implantation volumes per center and operator experience, which could have influenced complication rates.

## 6. Conclusions

This study showed that there was a statistically significant increase in complications associated with dual-lead PM implantations compared to single-lead PM procedures (3.27% vs. 2.85%, *p* < 0.001). However, no such difference was observed for ICD implantations (1.46% vs. 1.39%, *p* = 0.635). Lead dislocations emerged as the most frequent complication in both new PM and ICD implantations. Although perioperative mortality was low overall, it increased significantly in cases of intrahospital complications, with a 2.5-fold increase in PM implantations and an 8-fold increase in ICD implantations. We identified independent risk factors that may guide future implantation procedures and improve patient safety.

## Figures and Tables

**Table 1:** Perioperative complications in PM-Group: *p*_/2_ < 0.001; Odds Ratio 1.154 (95%KI: 1.096–1.426).

Pacemaker		**Perioperative Complications**	
	**Total**	**No**	**Yes**
Single Lead	29,852 (21.76%)	29,002 (97.15%)	850 (2.85%)
Dual Lead	107,356 (78.24%)	103,844 (96.73%)	3512 (3.27%)
Total	137,208	132,846 (96.83%)	4362 (3.17%)

**Table 2:** Perioperative complications in ICD-Group: *p*_/2_ = 0.635; Odds Ratio 1.046 (95%KI: 0.869–1.260).

ICD		**Perioperative Complications**	
	**Total**	**No**	**Yes**
Single Lead	18,743 (57.96%)	18,482 (98.61%)	261 (1.39%)
Dual Lead	13,596 (42.04%)	13,398 (98.54%)	198 (1.46%)
Total	32,339	31,880 (98.58%)	459 (1.42%)

**Table 3:** PM—Intrahospital complications: Incidence (*VDD).

		PM—Lead		
	Total	Single	Dual	*p*
Perioperative complications (incidence in %)	4362 (3.2)	850 (2.8)	3.512 (3.3)	<0.001
Asystole, *n* (%)	189 (0.1)	49 (0.2)	140 (0.1)	0.18
Ventricular fibrillation, *n* (%)	29 (0.0)	7 (0.0)	22 (0.0)	0.82
Pneumothorax, *n* (%)	718 (0.5)	160 (0.5)	558 (0.5)	0.72
Hemothorax, *n* (%)	50 (0.0)	4 (0.0)	46 (0.0)	0.016
Pericardial effusion/tamponade, *n* (%)	161 (0.1)	17 (0.1)	144 (0.1)	0.001
Lead dislocation, *n* (%)	2408 (1.8)	406 (1.4)	2002 (1.9)	<0.001
Atrial lead dislocation, *n* (%)	1259 (0.9)	0 (0.0)	1259 (1.2)	<0.001
Ventricular lead dislocation, *n* (%)	1212 (0.9)	406 (1.4)	806 (0.8)	<0.001
Lead dysfunction, *n* (%)	583 (0.4)	136 (0.5)	447 (0.4)	0.37
Atrial lead dysfunction, *n* (%)	91 (0.1)	1 * (0.0)	90 (0.1)	<0.001
Ventricular lead dysfunction, *n* (%)	528 (0.4)	135 (0.5)	393 (0.4)	0.038
Pocket hematoma, *n* (%)	282 (0.2)	104 (0.3)	178 (0.2)	<0.001
Wound infection, *n* (%)	9 (0.0)	2 (0.0)	7 (0.0)	1.00
Other, *n* (%)	278 (0.2)	19 (0.1)	259 (0.2)	<0.001

* in VDD-PM implantation.

**Table 4:** ICD—Intrahospital complications: Incidence (*VDD).

		ICD—Lead		
	Total	Single	Dual	*p*
Perioperative complications (incidence in %)	459 (1.4)	261 (1.4)	198 (1.5)	0.64
Cardiopulmonary resuscitation (CPR), *n* (%)	19 (0.1)	14 (0.1)	5 (0.0)	0.24
Pneumothorax, *n* (%)	114 (0.4)	69 (0.4)	45 (0.3)	0.63
Hemothorax, *n* (%)	35 (0.1)	31 (0.2)	4 (0.0)	<0.001
Pericardial effusion/tamponade, *n* (%)	9 (0.0)	2 (0.0)	7 (0.1)	0.041
Lead dislocation, *n* (%)	145 (0.4)	72 (0.4)	73 (0.5)	0.043
Atrial lead dislocation, *n* (%)	52 (0.2)	0 (0.0)	52 (0.4)	<0.001
Ventricular lead dislocation, *n* (%)	95 (0.3)	72 (0.4)	23 (0.2)	<0.001
Lead dysfunction, *n* (%)	22 (0.1)	12 (0.1)	10 (0.1)	0.83
Atrial lead dysfunction, *n* (%)	5 (0.0)	4 * (0.0)	1 (0.0)	0.41
Ventricular lead dysfunction, *n* (%)	17 (0.1)	8 (0.0)	9 (0.0)	0.46
Pocket hematoma, *n* (%)	69 (0.2)	55 (0.3)	14 (0.1)	<0.001
Wound infection, *n* (%)	8 (0.0)	4 (0.0)	8 (0.0)	0.73
Other, *n* (%)	54 (0.2)	10 (0.1)	44 (0.3)	<0.001

* in VDD-ICD implantation.

**Table 5:** PM—Discharge reason and Mortality.

PM—Discharge Reason		Perioperative Complication		
	All	No	Yes	*p*
Discharge/Transfer, *n* (%)	134,877 (98.3)	130,712 (96.9)	4165 (95.5)	<0.001
Mortality, *n* (%)	2331 (1.7)	2134 (1.6)	197 (4.5)	<0.001

**Table 6:** ICD—Discharge reason and Mortality.

ICD—Discharge Reason		Perioperative Complication		
	All	No	Yes	*p*
Discharge/Transfer, n (%)	31.734 (99.5)	31.734 (99.5)	441 (96.1)	<0.001
Mortality, *n* (%)	164 (0.5)	146 (0.5)	18 (3.9)	<0.001

## Data Availability

The datasets presented in this article are not readily available due to data protection policies enforced by QS-NRW. Requests to access the datasets should be directed to QS-NRW.

## Supplementary Materials

The following supporting information can be downloaded at: https://www.mdpi.com/article/10.3390/jcm14093203/s1, Table S1: PM—Prevalence of Subgroups and intrahospital complications (Incidence); Table S2: ICD—Prevalence of Subgroups and intrahospital complications (Incidence).

## References

[1] IQTIG (2021). Federal Evaluation for the Data Collection Year 2020 (Germany)—Pacemaker Implantations: Quality Indicators and Key Figures.

[2] IQTIG (2021). Federal Evaluation for the Data Collection Year 2020 (Germany)—Implantable Defibrillator Implantations Quality Indicators and Key Figures.

[3] Chauhan A., Grace A.A., Newell S.A., Stone D.L., Shapiro L.M., Schofield P.M., Petch M.C. (1994). Early complications after dual chamber versus single chamber pacemaker implantation. Pacing Clin. Electrophysiol..

[4] Cantillon D.J., Exner D.V., Badie N., Davis K., Gu N.Y., Nabutovsky Y., Doshi R. (2017). Complications and Health Care Costs Associated with Transvenous Cardiac Pacemakers in a Nationwide Assessment. JACC Clin. Electrophysiol..

[5] Greenspon A.J., Patel J.D., Lau E., Ochoa J.A., Frisch D.R., Ho R.T., Pavri B.B., Kurtz S.M. (2012). Trends in Permanent Pacemaker Implantation in the United States from 1993 to 2009: Increasing Complexity of Patients and Procedures. J. Am. Coll. Cardiol..

[6] Lamas G.A., Lee K.L., Sweeney M.O., Silverman R., Leon A., Yee R., Marinchak R.A., Flaker G., Schron E., Orav E.J. (2002). Ventricular Pacing or Dual-Chamber Pacing for Sinus-Node Dysfunction. N. Engl. J. Med..

[7] Johansen J.B. (2015). Danish Pacemaker and ICD Register Annual Report 2015.

[8] Link M.S., Estes N.A., Griffin J.J., Wang P.J., Maloney J.D., Kirchhoffer J.B., Mitchell G.F., Orav J., Goldman L., Lamas G.A. (1998). Complications of dual chamber pacemaker implantation in the elderly. Pacemaker Selection in the Elderly (PASE) Investigators. J. Interv. Card. Electrophysiol..

[9] Kiviniemi M.S., Pirnes M.A., Eränen H.J., Kettunen R.V., Hartikainen J.E. (1999). Complications related to permanent pacemaker therapy. Pacing Clin. Electrophysiol..

[10] Udo E.O., Zuithoff N.P.A., van Hemel N.M., de Cock C.C., Hendriks T., Doevendans P.A., Moons K.G.M. (2012). Incidence and predictors of short- and long-term complications in pacemaker therapy: The FOLLOWPACE study. Heart Rhythm.

[11] van Eck J.W., van Hemel N.M., Zuithof P., van Asseldonk J.P., Voskuil T.L., Grobbee D.E., Moons K.G. (2007). Incidence and predictors of in-hospital events after first implantation of pacemakers. Europace.

[12] Opić P., van Kranenburg M., Yap S.C., van Dijk A.P., Budts W., Vliegen H.W., van Erven L., Can A., Sahin G., Theuns D.A. (2013). Complications of pacemaker therapy in adults with congenital heart disease: A multicenter study. Int. J. Cardiol..

[13] Shakya S., Matsui H., Fushimi K., Yasunaga H. (2017). In-hospital complications after implantation of cardiac implantable electronic devices: Analysis of a national inpatient database in Japan. J. Cardiol..

[14] Aggarwal R.K., Connelly D.T., Ray S.G., Ball J., Charles R.G. (1995). Early complications of permanent pacemaker implantation: No difference between dual and single chamber systems. Br. Heart J..

[15] Eberhardt F., Bode F., Bonnemeier H., Boguschewski F., Schlei M., Peters W., Wiegand U.K. (2005). Long term complications in single and dual chamber pacing are influenced by surgical experience and patient morbidity. Heart.

[16] Ellenbogen K.A., Hellkamp A.S., Wilkoff B.L., Camunãs J.L., Love J.C., Hadjis T.A., Lee K.L., Lamas G.A. (2003). Complications arising after implantation of DDD pacemakers: The MOST experience. Am. J. Cardiol..

[17] Mueller X., Sadeghi H., Kappenberger L. (1990). Complications after single versus dual chamber pacemaker implantation. Pacing Clin. Electrophysiol..

[18] Al-Khatib S.M., Greiner M.A., Peterson E.D., Hernandez A.F., Schulman K.A., Curtis L.H. (2008). Patient and implanting physician factors associated with mortality and complications after implantable cardioverter-defibrillator implantation, 2002–2005. Circ. Arrhythm. Electrophysiol..

[19] Al-Khatib S.M., Lucas F.L., Jollis J.G., Malenka D.J., Wennberg D.E. (2005). The Relation Between Patients’ Outcomes and the Volume of Cardioverter-Defibrillator Implantation Procedures Performed by Physicians Treating Medicare Beneficiaries. J. Am. Coll. Cardiol..

[20] Bardy G.H., Lee K.L., Mark D.B., Poole J.E., Packer D.L., Boineau R., Domanski M., Troutman C., Anderson J., Johnson G. (2005). Amiodarone or an implantable cardioverter-defibrillator for congestive heart failure. N. Engl. J. Med..

[21] Bogossian H., Frommeyer G., Hochadel M., Ince H., Spitzer S.G., Eckardt L., Maier S.K.G., Kleemann T., Brachmann J., Stellbrink C. (2020). Single chamber implantable cardioverter defibrillator compared to dual chamber implantable cardioverter defibrillator: Less is more! Data from the German Device Registry. Clin. Res. Cardiol..

[22] DiMarco J.P. (2003). Implantable cardioverter-defibrillators. N. Engl. J. Med..

[23] Gold M.R., Peters R.W., Johnson J.W., Shorofsky S.R. (1996). Complications associated with pectoral cardioverter-defibrillator implantation: Comparison of subcutaneous and submuscular approaches. Worldwide Jewel Investigators. J. Am. Coll. Cardiol..

[24] Gould P.A. (2006). Complications Associated with Implantable Cardioverter-Defibrillator Replacement in Response to Device Advisories. JAMA.

[25] Haines D.E., Wang Y., Curtis J. (2011). Implantable Cardioverter-Defibrillator Registry Risk Score Models for Acute Procedural Complications or Death After Implantable Cardioverter-Defibrillator Implantation. Circulation.

[26] Hlatky M.A., Saynina O., McDonald K.M., Garber A.M., McClellan M.B. (2002). Utilization and outcomes of the implantable cardioverter defibrillator, 1987 to 1995. Am. Heart J..

[27] Kadish A., Dyer A., Daubert J.P., Quigg R., Estes N.A.M., Anderson K.P., Calkins H., Hoch D., Goldberger J., Shalaby A. (2004). Prophylactic Defibrillator Implantation in Patients with Nonischemic Dilated Cardiomyopathy. N. Engl. J. Med..

[28] Koneru J.N., Jones P.W., Hammill E.F., Wold N., Ellenbogen K.A. (2018). Risk Factors and Temporal Trends of Complications Associated with Transvenous Implantable Cardiac Defibrillator Leads. J. Am. Heart Assoc..

[29] Kramer D.B., Kennedy K.F., Noseworthy P.A., Buxton A.E., Josephson M.E., Normand S.-L., Spertus J.A., Zimetbaum P.J., Reynolds M.R., Mitchell S.L. (2013). Characteristics and Outcomes of Patients Receiving New and Replacement Implantable Cardioverter-Defibrillators. Circ. Cardiovasc. Qual. Outcomes.

[30] Kron J., Herre J., Renfroe E.G., Rizo-Patron C., Raitt M., Halperin B., Gold M., Goldner B., Wathen M., Wilkoff B. (2001). Lead- and device-related complications in the antiarrhythmics versus implantable defibrillators trial. Am. Heart J..

[31] Lee D.S., Krahn A.D., Healey J.S., Birnie D., Crystal E., Dorian P., Simpson C.S., Khaykin Y., Cameron D., Janmohamed A. (2010). Evaluation of Early Complications Related to De Novo Cardioverter Defibrillator Implantation. J. Am. Coll. Cardiol..

[32] Moss A.J., Zareba W., Hall W.J., Klein H., Wilber D.J., Cannom D.S., Daubert J.P., Higgins S.L., Brown M.W., Andrews M.L. (2002). Prophylactic implantation of a defibrillator in patients with myocardial infarction and reduced ejection fraction. N. Engl. J. Med..

[33] Pavia S., Wilkoff B. (2001). The management of surgical complications of pacemaker and implantable cardioverter-defibrillators. Curr. Opin. Cardiol..

[34] Reynolds M.R., Cohen D.J., Kugelmass A.D., Brown P.P., Becker E.R., Culler S.D., Simon A.W. (2006). The frequency and incremental cost of major complications among medicare beneficiaries receiving implantable cardioverter-defibrillators. J. Am. Coll. Cardiol..

[35] Rosenqvist M.R., Beyer T., Block M., Den Dulk K., Minten J., Lindemans F. (1998). Adverse Events with Transvenous Implantable Cardioverter-Defibrillators. Circulation.

[36] Tsai V., Goldstein M.K., Hsia H.H., Wang Y., Curtis J., Heidenreich P.A. (2011). Influence of Age on Perioperative Complications Among Patients Undergoing Implantable Cardioverter-Defibrillators for Primary Prevention in the United States. Circ. Cardiovasc. Qual. Outcomes.

[37] Zhan C., Baine W.B., Sedrakyan A., Steiner C. (2008). Cardiac Device Implantation in the United States from 1997 through 2004: A Population-based Analysis. J. Gen. Intern. Med..

[38] Almendral J., Arribas F., Wolpert C., Ricci R., Adragao P., Cobo E., Navarro X., Quesada A. (2008). Dual-chamber defibrillators reduce clinically significant adverse events compared with single-chamber devices: Results from the DATAS (Dual chamber and Atrial Tachyarrhythmias Adverse events Study) trial. Europace.

[39] Cheng A., Wang Y., Curtis J.P., Varosy P.D. (2010). Acute Lead Dislodgements and In-Hospital Mortality in Patients Enrolled in the National Cardiovascular Data Registry Implantable Cardioverter Defibrillator Registry. J. Am. Coll. Cardiol..

[40] Defaye P., Boveda S., Klug D., Beganton F., Piot O., Narayanan K., Périer M.C., Gras D., Fauchier L., Bordachar P. (2017). Dual- vs. single-chamber defibrillators for primary prevention of sudden cardiac death: Long-term follow-up of the Défibrillateur Automatique Implantable-Prévention Primaire registry. Europace.

[41] Dewland T.A., Pellegrini C.N., Wang Y., Marcus G.M., Keung E., Varosy P.D. (2011). Dual-Chamber Implantable Cardioverter-Defibrillator Selection Is Associated with Increased Complication Rates and Mortality Among Patients Enrolled in the NCDR Implantable Cardioverter-Defibrillator Registry. J. Am. Coll. Cardiol..

[42] Freeman J.V., Wang Y., Curtis J.P., Heidenreich P.A., Hlatky M.A. (2012). Physician Procedure Volume and Complications of Cardioverter-Defibrillator Implantation. Circulation.

[43] Hsu J.C., Varosy P.D., Bao H., Wang Y., Curtis J.P., Marcus G.M. (2012). Low Body Mass Index but Not Obesity Is Associated with In-Hospital Adverse Events and Mortality Among Implantable Cardioverter-Defibrillator Recipients: Insights from the National Cardiovascular Data Registry. J. Am. Heart Assoc..

[44] Takahashi T., Bhandari A.K., Watanuki M., Cannom D.S., Sakurada H., Hiraoka M. (2002). High Incidence of Device-Related and Lead-Related Complications in the Dual-Chamber Implantable Cardioverter Defibrillator Compared with the Single-Chamber Version. Circ. J..

[45] Ueda A., Oginosawa Y., Soejima K., Abe H., Kohno R., Ohe H., Momose Y., Nagaoka M., Matsushita N., Hoshida K. (2016). Outcomes of single- or dual-chamber implantable cardioverter defibrillator systems in Japanese patients. J. Arrhythm..

[46] Glikson M., Nielsen J.C., Kronborg M.B., Michowitz Y., Auricchio A., Barbash I.M., Barrabés J.A., Boriani G., Braunschweig F., Brignole M. (2021). 2021 ESC Guidelines on cardiac pacing and cardiac resynchronization therapy. Eur. Heart J..

[47] Wilkoff B.L., Fauchier L., Stiles M.K., Morillo C.A., Al-Khatib S.M., Almendral J., Aguinaga L., Berger R.D., Cuesta A., Daubert J.P. (2016). 2015 HRS/EHRA/APHRS/SOLAECE expert consensus statement on optimal implantable cardioverter-defibrillator programming and testing. Heart Rhythm.

[48] Carlsson J., Erdogan A., Neuzner J., Fröhlig G. (2020). Defibrillator. Herzschrittmacher- und Defibrillator-Therapie.

[49] Tobin K., Stewart J., Westveer D., Frumin H. (2000). Acute complications of permanent pacemaker implantation: Their financial implication and relation to volume and operator experience. Am. J. Cardiol..

[50] Pakarinen S., Oikarinen L., Toivonen L. (2010). Short-term implantation-related complications of cardiac rhythm management device therapy: A retrospective single-centre 1-year survey. Europace.

[51] Wiegand U.K., LeJeune D., Boguschewski F., Bonnemeier H., Eberhardt F., Schunkert H., Bode F. (2004). Pocket hematoma after pacemaker or implantable cardioverter defibrillator surgery: Influence of patient morbidity, operation strategy, and perioperative antiplatelet/anticoagulation therapy. Chest.

[52] Nowak B., Misselwitz B., Erdogan A., Funck R., Irnich W., Israel C.W., Olbrich H.G., Schmidt H., Sperzel J., Zegelman M. (2010). Do gender differences exist in pacemaker implantation?--results of an obligatory external quality control program. Europace.

[53] Kirkfeldt R.E., Johansen J.B., Nohr E.A., Jørgensen O.D., Nielsen J.C. (2014). Complications after cardiac implantable electronic device implantations: An analysis of a complete, nationwide cohort in Denmark. Eur. Heart J..

[54] Humphries K.H., Hawkins N. (2020). Sex Differences in Complications and Outcomes of Cardiac Implantable Electronic Devices: Time to Evaluate Our Practice. Can. J. Cardiol..

[55] Mohamed M.O., Volgman A.S., Contractor T., Sharma P.S., Kwok C.S., Rashid M., Martin G.P., Barker D., Patwala A., Mamas M.A. (2020). Trends of Sex Differences in Outcomes of Cardiac Electronic Device Implantations in the United States. Can. J. Cardiol..

[56] Nowak B., Tasche K., Barnewold L., Heller G., Schmidt B., Bordignon S., Chun K.R., Fürnkranz A., Mehta R.H. (2015). Association between hospital procedure volume and early complications after pacemaker implantation: Results from a large, unselected, contemporary cohort of the German nationwide obligatory external quality assurance programme. Europace.

[57] Chen B.-W., Liu Q., Wang X., Dang A.-M. (2014). Are dual-chamber implantable cardioverter-defibrillators really better than single-chamber ones? A systematic review and meta-analysis. J. Interv. Card. Electrophysiol..

[58] Hu Z.-Y., Zhang J., Xu Z.-T., Gao X.-F., Zhang H., Pan C., Chen S.-L. (2016). Efficiencies and Complications of Dual Chamber versus Single Chamber Implantable Cardioverter Defibrillators in Secondary Sudden Cardiac Death Prevention: A Meta-analysis. Heart Lung Circ..

[59] Mei D.A., Imberti J.F., Vitolo M., Bonini N., Gerra L., Romiti G.F., Proietti M., Lip G.Y.H., Boriani G. (2023). Single-lead VDD pacing: A literature review on short-term and long-term performance. Expert Rev. Med. Devices.

[60] Peterson P.N., Daugherty S.L., Wang Y., Vidaillet H.J., Heidenreich P.A., Curtis J.P., Masoudi F.A. (2009). Gender Differences in Procedure-Related Adverse Events in Patients Receiving Implantable Cardioverter-Defibrillator Therapy. Circulation.

[61] Pires L.A., Sethuraman B., Guduguntla V.D., Todd K.M., Yamasaki H., Ravi S. (2002). Outcome of women versus men with ventricular tachyarrhythmias treated with the implantable cardioverter defibrillator. J. Cardiovasc. Electrophysiol..

[62] Russo A.M., Day J.D., Stolen K., Mullin C.M., Doraiswamy V., Lerew D.L., Olshansky B. (2009). Implantable cardioverter defibrillators: Do women fare worse than men? Gender comparison in the INTRINSIC RV trial. J. Cardiovasc. Electrophysiol..

[63] Mei D.A., Imberti J.F., Vitolo M., Bonini N., Serafini K., Mantovani M., Tartaglia E., Birtolo C., Zuin M., Bertini M. (2024). Systematic review and meta-analysis on the impact on outcomes of device algorithms for minimizing right ventricular pacing. EP Europace.

[64] Kichloo A., Shaka H., Aljadah M., Amir R., Albosta M., Jamal S., Khan M.Z., Wani F., Mir K.M., Kanjwal K. (2021). Predictors of outcomes in hospitalized patients undergoing pacemaker insertion: Analysis from the national inpatient database (2016–2017). Pacing Clin. Electrophysiol..

[65] Mahapatra S., Bybee K.A., Bunch T.J., Espinosa R.E., Sinak L.J., McGoon M.D., Hayes D.L. (2005). Incidence and predictors of cardiac perforation after permanent pacemaker placement. Heart Rhythm.

[66] Hasan F., Nedios S., Karosiene Z., Scholten M., Lemke B., Tulka S., Knippschild S., Macher-Heidrich S., Adomeit H.J., Zarse M. (2022). Perioperative complications after pacemaker implantation: Higher complication rates with subclavian vein puncture than with cephalic vein cutdown. J. Interv. Card. Electrophysiol..

[67] Nowak B., Misselwitz B., Przibille O., Mehta R.H. (2016). Is mortality a useful parameter for public reporting in pacemaker implantation? Results of an obligatory external quality control programme. Europace.

[68] Mandawat A., Curtis J.P., Mandawat A., Njike V.Y., Lampert R. (2013). Safety of Pacemaker Implantation in Nonagenarians. Circulation.

[69] Bogossian H., Panteloglou D., Karosiene Z., Macher-Heidrich S., Adomeit H.J., Lemke B., Israel C.W. (2021). Peripoperative Mortalität nach ICD-Implantation. Herz.

[70] Imberti J.F., Mei D.A., Fontanesi R., Gerra L., Bonini N., Vitolo M., Turco V., Casali E., Boriani G. (2023). Low Occurrence of Infections and Death in a Real-World Cohort of Patients with Cardiac Implantable Electronic Devices. J. Clin. Med..

