# Supplementary material for: Comparison of Single- vs. Dual-Lead CIEDs Regarding Perioperative Complications-Analysis of the Quality Assurance Data of the State of North Rhine-Westphalia, Germany
Source: J Clin Med. 2025 May 6;14(9):3203. doi: 10.3390/jcm14093203 (PMC12072410; doi:10.3390/jcm14093203)
Supplement: Supplementary file 1 [file jcm-14-03203-s001.zip › jcm-3496503-supplementary.pdf]

Supplementary Table S1. PM - Prevalence of Subgroups and intrahospital complications (Incidence)

PM	Total	Complication		p-Value
		No	Yes	
**Gender**				
Women, n (%)	66.030 (48.1)	63.643 (96,4)	2.387 (3.6)	<0.001
**Age**				
<20, n (%)	27	27	0	1.00
20-29, n (%)	306 (0.2)	272 (88.9)	34 (11.1)	<0.001
30-39, n (%)	474 (0.3)	465 (98.1)	9 (1.9)	0.15
40-49, n (%)	2.084 (1.5)	2.043 (98.0)	41 (2.0)	0.001
50-59, n (%)	5.222 (3.8)	5.047 (96.6)	175 (3.4)	0.47
60-69, n (%)	16.014 (11.7)	15.402 (96.2)	612 (3.8)	<0.001
70-79, n (%)	53.237 (38.8)	51.641 (97.0)	1.596 (3.0)	0.02
80-89, n (%)	52.694 (38.4)	50.914 (96.6)	1.780 (3.4)	0.01
90-99, n (%)	7.085 (5.2)	6.970 (98.4)	115 (1.6)	0.001
100-102	65	65	0	0.27
**Left ventricular ejection fraction**				
unknown, n (%)	15.004 (10.9)	14.661 (97.7)	343 (3.3)	<0.001
LVEF >50, n (%)	107.020 (78.0)	103.474 (96.7)	3.546 (3.2)	<0.001
LVEF 35-50, n (%)	13.901 (10.1)	13.462 (96.8)	439 (2.7)	0.90
LVEF ≤35, n (%)	1.283 (0.9)	1.249 (97.3)	34 (2.9)	0.34
**PM System**				
AAI, n (%)	197 (0.1)	197	0	0.003
VVI, n (%)	29.029 (21.2)	28.197 (97.1)	832 (2.9)	0.001
DDD, n (%)	107.356 (78.2)	103.844 (96.4)	3512 (3.3)	<0.001
VDD, n (%)	626 (0.5)	608 (97.1)	18 (2.9)	0.81
**ASA Classifikation**				
ASA I - normal healthy patient, n (%)	9.253 (6.7)	90.13 (97.4)	240 (2.6)	0.001
ASA II - mild systemic disese, n (%)	61.083 (45.5)	58.941 (96.5)	2.142 (3.5)	<0.001
ASA III - serve systemic disease, n (%)	61.993 (45.2)	60.239 (97.2)	1.754 (2.8)	<0.001
ASA IV - ASA III + constant life threatening, n (%)	4.710 (3.4)	4.492 (95.4)	218 (4.6)	<0.001
ASA V - moribund patient, n (%)	169 (0.1)	161 (95.3)	8 (4.7)	0.27
**Main clinical symptom**				
non (asymtopmatic), n (%)	2339 (1.7)	2268 (97.0)	71 (3.0)	Not calculated *
Presyncope/Dizziness, n (%)	63055 (46.0)	60994 (96.7)	2061 (3.3)	
Syncope, single, n (%)	16906 (12.3)	16371 (96.8)	535 (3.2)	
Syncope, recurrent, n (%)	38247 (27.9)	36954 (96.6)	1293 (3.4)	
Syncope with injury, n (%)	5591 (4.1)	5405 (96.7)	186 (3.3)	
Heart insufficiency, NYHA II, n (%)	2603 (1.9)	2562 (98.4)	41 (1.6)	

Heart insufficiency, NYHA >II, n (%)	4725 (3.4)	4636 (98.1)	89 (1.9)	
Other, n (%)	3742 (2.7)	3656 (97.7)	86 (2.3)	
**Main indication**				
AVB °I, n (%)	395 (0.3)	389 (98.5)	6 (1.5)	0.06
AVB °II Wenckebach, n (%)	1510 (1.1)	1496 (99.1)	14 (0.9)	<0.001
AVB °II Mobitz, n (%)	15130 (11.0)	14578 (96.4)	552 (3.6)	<0.001
AVB °III, n (%)	35623 (26.0)	34548 (97.0)	1075 (3.0)	0.04
faszikuläre Leitungsstörung, n (%)	2607 (1.9)	2537 (97.3)	70 (2.7)	0.16
SSS/BTS Syndrome, n (%)	56043 (40.8)	54078 (96.5)	1965 (3.5)	<0.001
Bardycardiac Atrial fibrillation (AF), n (%)	22892 (16.7)	22342 (97.6)	550 (2.4)	<0.001
Carotis-Sinus-Syndrome (CSS), n (%)	939 (0.7)	878 (93.5)	61 (6.5)	<0.001
Vasovagale Syndrome (VVS), n (%)	324 (0.2)	320 (98.8)	4 (1.2)	0.039
Other	1739 (1.3)	1674 (96.3)	65 (3.7)	0.039
**Atrial rhythm**				
Sinus rhythmus, n (%)	40.052 (29.2)	38.673 (96.6)	1.379 (3.4)	<0.001
SR-Bradykardic/SA-Block, n (%)	39.846 (29.0)	38.473 (96.6)	1.373 (3.4)	<0.001
Parox./pers. AF or AFL, n (%)	11.647 (8.5)	11.223 (96.4)	424 (3.6)	0.003
Perm AF, n (%)	25.524 (18.6)	24.959 (97.8)	565 (2.2)	<0.001
Brady-Tachy-Syndrom, n (%)	19.575 (14.3)	18.967 (96.9)	608 (3.1)	0.54
Other, n (%)	564 (0.4)	551 (97.7)	13 (2.3)	0.28
**AV conduction**				
Normal AV conduction, n (%)	57.888 (42.2)	55.959 (96.7)	1.929 (3.3)	0.006
AV-B °I ≤300ms, n (%)	6.721 (4.9)	6.536 (97.2)	185 (2.8)	0.044
AV-B °I >300ms, n (%)	1.319 (1.0)	1.277 (96.8)	42 (3.2)	0.95
AV-B °II Wenckebach, n (%)	2.730 (2.0)	2.684 (98.3)	46 (1.7)	<0.001
AV-B °II Mobitz, n (%)	16.815 (12.3)	16.218 (96.4)	597 (3.6)	0.004
AV-B °III, n (%)	37.919 (27.6)	36.724 (96.8)	1.195 (3.2)	0.76
Not assessable (AF), n (%)	13.816 (10.1)	13.448 (97.3)	368 (2.7)	<0.001
**Intraventricular conduction**				
QRS <120ms, n (%)	115.134 (83,9)	111.370 (96.7)	3.764 (86,3)	<0.001
RBBB, n (%)	5.944 (4,3)	5.769 (97.1)	175 (4,0)	0.308
LAHB & RBBB, n (%)	4.816 (3,5)	4.696 (97.5)	120 (2,8)	0.006
LPHB & RBBB, n (%)	755 (0,6)	754 (99.9)	1 (0,0)	<0.001
LBBB, QRS 120-150ms, n (%)	3.632 (2,6)	3.576 (98.5)	56 (1,3)	<0.001
LBBB, QRS ≥150ms, n (%)	2.197 (1,6)	2.067 (94.1)	130 (3,0)	<0.001
Alternating BBB, n (%)	834 (0,6)	824 (98.8)	10 (0,2)	0.001
QRS ≥120ms, non-specific, n (%)	1.865 (1,4)	1.822 (97.7)	43 (1,0)	0.031
Other, n (%)	2.031 (1,8)	1.968 (96.9)	63 (1,7)	0.891

*not calculated because of multiple possible etiologies.

Supplementary Table S2. ICD - Prevalence of Subgroups and intrahospital complications (Incidence).

ICD	Total	Complication		p-Value
		No	Yes	
**Gender**				
Women, n (%)	7.062 (21.8)	6.938 (98.2)	124 (1.8)	0.008
**Age**				
<20, n (%)	99 (0.3)	99	0	0,41
20-29, n (%)	355 (1.1)	354 (99.7)	1 (0,3)	0,07
30-39, n (%)	541 (1.7)	535 (98.9)	6 (1,1)	0,71
40-49, n (%)	2.003 (6.2)	1.960 (97.9)	43 (2.1)	0,006
50-59, n (%)	5.818 (18.0)	5.757 (99.0)	61 (1.0)	0,008
60-69, n (%)	8.057 (24.9)	7.992 (99.2)	65 (0.8)	<0,001
70-79, n (%)	12.068 (37.3)	11.858 (98.3)	210 (1.7)	<0,001
80-89, n (%)	3385 (10.5)	3.313 (97.9)	72 (2.1)	<0,001
90-99, n (%)	13 (0.0)	12 (92.3)	1 (7.7)	0.170
**Left ventricular ejection fraction**				
unknown, n (%)	167 (0.5)	164 (98.2)	3 (1.8)	0,52
LVEF >50, n (%)	3.613 (11.2)	3.585 (99.2)	28 (0.8)	<0,001
LVEF 35-50, n (%)	3.786 (11.7)	3.731 (98.5)	55 (1.5)	0,83
LVEF ≤35, n (%)	24.773 (76.6%)	24.400 (98.5)	373 (1.5)	0,02
**ICD System**				
VVI, n (%)	18.344 (56.8)	18.114 (98.1)	230 (1.3)	0,004
DDD, n (%)	13.596 (42.0)	13.398 (98.5)	198 (1.5)	0,64
VDD, n (%)	399 (1.2)	368 (92.1)	31 (7.8)	<0,001
**ASA Classifikation**				
ASA I - normal healthy patient, n (%)	613 (1.9)	599 (97.7)	14 (2.3)	0,08
ASA II - mild systemic disese, n (%)	6.889 (21.3)	6.778 (98.4)	111 (1.6)	0,13
ASA III - serve systemic disease, n (%)	22.224 (68.7)	21.937 (98.7)	287 (1.3)	0,004
ASA IV - ASA III + constant life threatening, n (%)	2.609 (8.1)	2.563 (98.2)	46 (1.8)	0,12
ASA V - moribund patient, n (%)	4 (0.0)	3 (75.0)	1 (25.0)	0,06
**Main indication**				
Ventricular fibrillation, n (%)	4731 (14.6)	4673 (98.8)	58 (1.2)	0,26
VT, sustained (sVT, >30 sec) , n (%)	3806 (11.8)	3770 (99.1)	36 (0.9)	0,008
VT, non-sustained (nsVT, ≤ 30 sec) , n (%)	2103 (6.5)	2095 (99.6)	8 (0.4)	<0,001
Syncope, but no ECG, n (%)	1210 (3.7)	1193 (98.6)	17 (1.4)	0,99
No event (primary prevention) , n (%)	20000 (61.8)	19665 (98.3)	335 (1.7)	<0,001
Other, n (%)	489 (1.5)	484 (99.0)	5 (1,0)	0,57
**Main clinical symptoms**				
No symptoms, n (%)	866 (2.7)	861 (99.4)	5 (0.6)	
Cardiac arrest (CPR) , n (%)	5236 (16.2)	5173 (98.8)	63 (1.2)	
Cardiogenic shock, n (%)	448 (1.4)	438 (97.8)	10 (2,2)	
Lung edema, n (%)	249 (0.8)	249	0	Not
Syncope, n (%)	2820 (8.7)	2790 (98.9)	30 (1.1)	

Presyncope, n (%)	1566 (4.8)	1558 (99.5)	8 (0.5)	calculated*
Low blood pressure (e.g. <80mmHg), n (%)	407 (1.3)	403 (99.0)	4 (1.0)	
Angina pectoris, n (%)	235 (0.7)	235	0	
Other, n (%)	512 (1.6)	508 (99.2)	4 (0.8)	
No event (primary prevention), n (%)	20000 (61.8)	19665 (98.3)	335 (1.7)	
**Atrial rhythm**				
Sinus rhythmus, n (%)	20.307 (62.8)	20.027 (98.6)	280 (1.4)	0,44
SR-Bradykardie/SA-Block, n (%)	3.577 (11.1)	3.521 (98.4)	56 (1.6)	0,41
Parox./pers. AF or AFL, n (%)	2.557 (7.9)	2.502 (97.8)	55 (2.2)	0,002
Perm AF, n (%)	4.425 (13.7)	4.392 (99.3)	33 (0.7)	<0,001
Brady-Tachy-Syndrom, n (%)	1.424 (4.4)	1.389 (97.5)	35 (2.5)	0,001
Other, n (%)	49 (0.2)	49	0	1,0
**AV conduction**				
Normal AV conduction, n (%)	27.140 (83.9)	26.719 (98.4)	421 (1.6)	<0,001
AV-B °I ≤300ms, n (%)	2.016 (6.2)	2.007 (99.6)	9 (0.4)	<0,001
AV-B °I >300ms, n (%)	99 (0.3)	99	0	0,41
AV-B °II Wenckebach, n (%)	176 (0.5)	176	0	0,19
AV-B °II Mobitz, n (%)	380 (1.2)	375 (98.7)	5 (1,3)	1,0
AV-B °III, n (%)	801 (2.5)	786 (98.6)	15 (1.9)	1,0
Not assessable (AF), n (%)	1.727 (5.3)	1.718 (99.5)	9 (0,5)	0,001
**Intraventricular conduction**				
QRS <120ms, n (%)	28.879 (89.3)	28.487 (98.6)	398 (1.4)	0,08
RBBB, n (%)	930 (2.9)	901 (96.9)	29 (3.1)	<0,001
LAHB & RBBB, n (%)	467 (1.4)	467	0	0,002
LPHB & RBBB, n (%)	14 (0.0)	14	0	1,0
LBBB, QRS 120-150ms, n (%)	1.029 (3.2)	1.019 (99.0)	10 (1.0)	0,28
LBBB, QRS ≥150ms, n (%)	411 (1.3)	397 (96.6)	14 (3.4)	0,002
Alternating BBB, n (%)	46 (0.1)	46	0	1,0
QRS ≥120ms, non-specific, n (%)	241 (0.7)	239 (99.2)	2 (0,8)	0,78
Other, n (%)	322 (1.0)	316 (98.1)	6 (1.9)	0,47

*not calculated because of multiple possible etiologies.
